# autopsych: An R Shiny tool for the reproducible Rasch analysis, differential item functioning, equating, and examination of group effects

**DOI:** 10.1371/journal.pone.0257682

**Published:** 2021-10-11

**Authors:** Matthew G. R. Courtney, Kevin C. T. Chang, Bing Mei, Kane Meissel, Luke I. Rowe, Laila B. Issayeva

**Affiliations:** 1 Graduate School of Education, Nazarbayev University, Nur-Sultan, Kazakhstan; 2 Department of Statistics, The University of Auckland, Auckland, New Zealand; 3 School of Foreign Languages, Henan University, Henan, China; 4 Faculty of Education and Social Work, The University of Auckland, Auckland, New Zealand; 5 National School of Education, Australian Catholic University, Melbourne, Australia; 6 Center for Pedagogical Measurements, Nazarbayev Intellectual Schools, Nur-Sultan, Kazakhstan; University of Copenhagen, DENMARK

## Abstract

In this paper, we present **autopsych**, a novel online tool that allows school assessment experts, test developers, and researchers to perform routine psychometric analyses and equating of student test data and to examine the effect of student demographic and group conditions on student test performance. The app extends current open-source software by providing (1) extensive embedded result narration and summaries for written reports, (2) improved handling of partial credit data via customizable item-person Wright maps, (3) customizable item- and person-flagging systems, (4) item-response theory model constraints and controls, (5) many-facets Rasch analysis to examine item bias, (6) Rasch fixed item equating for mapping student ability across test forms, (7) tabbed spreadsheet outputs and immediate options for secondary data analysis, (8) customizable graphical color schemes, (9) extended ANOVA analysis for examining group differences, and (10) inter-rater reliability analyses for the verifying the consistency of rater scoring systems. We present the app’s architecture and functionalities and test its performance with simulated and real-world small-, medium-, and large-scale assessment data. Implications and planned future developments are also discussed.

## 1 Introduction

Assessment plays a central role in society. While assessment is common to the educational sector, it is also important to clinical contexts and personnel selection. In educational contexts, particular physical, intellectual, and moral states are demanded of students by the unique political, social, and educational milieu for which they are placed [1]. Correspondingly, the responsibility for measuring student ability or knowledge in a valid, reliable, and unbiased way [2] rests on the shoulders of those administering the assessments.

Recent advancements in the field of psychometrics have enabled school assessment experts, test developers, and researchers access to user-friendly psychometric software interfaces (e.g., [3]). The app presented in this paper extends current open-source software with a general focus on the traditional Rasch-based approach [4, 5] to test validation. In this paper we provide a general background to classical test theory (CTT) and Rasch modelling before providing more specific details of the ways in which the autopsych app extends current open-source software capabilities. We finish by testing the app’s local and cloud-based performance under different data conditions, prior to providing some ideas for the app’s future development.

### 1.1 Classical test theory

Classical test theory (CTT) is a traditional approach to examining the quality of an exam or scale based on the students’ resultant data scoring patterns [6]. From within this framework, a question’s difficulty is estimated by the number of students that respond incorrectly to that item. Likewise, student ability is estimated by the items that items that are marked correct for that student. In CTT, the quality and function of a question are assessed by the correlation between the students’ response pattern for that item and the students’ total scores in the test. The item-rest correlation reveals the strength of the correlation between the item-score and the test score (without the focal item), while the bi- and poly-serial correlation coefficients correct for attenuation of the strength of the correlation due to the limited number of response options in dichotomous and polytomous data, respectively [7]. Here, item-total correlations reveal the degree to which items “discriminate” between students with positive numbers viewed favorably, though negative estimates considered problematic and generally necessary to remove. Though not necessarily problematic, item-rest correlations below *r* = .10 may be considered small and flagged as lower performing [8]. Upper and lower confidence intervals (CIs) can be estimated for each correlation as an additional metric that accounts for the size of the student sample. Instances where lower 95% CIs (or more strict CIs) are below zero, though not necessarily problematic, may also be flagged as lower performing [9]. In CTT, the Cronbach’s alpha reliability estimate is commonly employed to evaluate the overall internal consistency of a scale or test [10] with 0.50 = poor, 0.60 = questionable, 0.70 = acceptable, 0.80 = good, and 0.90 = excellent [11]. As an additional metric, the alpha reliability of the scale or test can also be examined upon removal of each respective item in the test. Where the reliability of the test improves (alpha increases) because of the removal of an item, though not necessarily problematic, provides another way to flag lower performing items in a test. The application of CTT provides a useful way to validate scales and tests. However, enabling users to automatically customize the flagging of items to their own specifications (i.e., item-rest correlations lower than a certain limit and the size of the CIs) would be useful.

### 1.2 Rasch modelling

The Rasch modelling framework was developed in the 1950s by the Danish mathematician, George Rasch. The framework was first applied to examine the quality of student achievement testing among Danish school children, and intelligence testing among Danish military personnel. Today, the framework is widely applied to the social sciences for analysis of traits and has also been adopted in clinical and public health research for the measurement of diverse outcomes [12].

The Rasch model provides a probabilistic interpretation of student competence. Such interpretations have been the basis for theoretical links between “assessment, teaching and learning, curriculum resources, and policy development” [13, p87]. In the case of dichotomous scoring outcomes (i.e., 0, incorrect, and, 1, correct), the Rasch model expresses student performance as,

$$P\left(X_{ni}=1|\theta_{n},\delta_{i}\right)=\frac{e^{D\left(\theta_{n}-\delta_{i}\right)}}{1+e^{D\left(\theta_{n}-\delta_{i}\right)}}$$ (1)

where, *θ*_*n*_ is the person ability, *δ*_*i*_ is item difficulty (reflecting, in a binary sense, “the nature of the trait”, (13, p87-88), *e* is the mathematical constant, and *D* is the scaling constant 1.702 for matching logistic and probit metrics very closely [14]; removed in subsequent formula for simplicity). To note, where *θ*_*n*_ = *δ*_*i*_, the probability of a student’s success on the item is *p* = .50, the point at which, conceivably, a student may be functioning within his or her zone of proximal development [13].

To account for non-binary conceptualizations of student proficiency (i.e., 0, 1, 2 scoring and rubric-based scoring), Rasch modelling was extended to account for partial credit scored test questions and polytomously-scored developmental criteria [15, 16]. Here, in a general sense, the dichotomous Rasch model was extended to account for polytomous scoring by representing each pair of adjacent scores as a string of ordered categories [16],

$$P\left(X_{nij}=1|\theta_{n},\delta_{ij}\right)=\frac{e^{\left(\theta_{n}-\delta_{ij}\right)}}{1+e^{\left(\theta_{n}-\delta_{ij}\right)}},\;j=1,\;2,\;\ldots ,\;m_{i}$$ (2)

where *j* specifies the step to be taken by person *n* in item *i* from the lower of the adjacent score category to the higher category. Specifically, instead of defining a single item response function for an item, the partial credit model (PCM) defines mi category-response functions for each item–each function representing the conditional probability of student *n* completing *j* steps in item *i* given that they complete either *j* − 1 of *j* [16].

Theorists [13] have argued that the sets of item criteria (or items) for which students have close to a .50 probability (50:50 odds; where *θ*_*n*_ = *δ*_*ij*_) of success “can be linked to research about the development of *human beings* (emphasis added) and the role that formal education plays in the process” [p90]. Specifically, this zone of proximal development (ZPD) can be defined as,

a state of readiness in which a student will be able to make certain kinds of conceptual connections, but not others; anything too simple for the student will quickly become boring; anything too difficult will quickly become demoralising.

[17 p122]

Here, it is argued that test scores (i.e., theta, *θ*_*n*_) should be interpreted as a starting point for instructional intervention, a ZPD point where students may be able to optimally improve with additional support. This reconceptualization of the test score as a point of intervention and instructional scaffolding can also be used to identify and build appropriate teaching resources and curricula policy [13, p90]. It has been common for these methodologies to be beyond the interest of the classroom teacher [4, p91]. This has been largely due to an inability to overcome the burden of purchasing proprietary software and the learning the programming language associated with learning how to use the software [18, p107].

However, today, psychometric software has become more user-friendly and ubiquitously accessible (see, for example [3]). Moreover, it is likely that schoolteachers with a background in Maths, Physics, Engineering, or Computer Science committed to improving the quality of assessments would be able to grasp and make use of such psychometric tools in an applied way—more certainly after being provided with a careful exposition of the fundamental principles and methodology.

### 1.3 Current psychometric software systems

Multiple psychometric software packages exist today and can be classified as either commercial with ongoing licenses [e.g., 19, 20] or non-commercial [e.g., 21, 22]. Currently, only several commercially available systems, such as XCalibre [23], offer automatic embedded narration, that is conditionally worded descriptive paragraphs of the results of the analysis in output technical reports. Currently, the freely available cloud-based ShinyItemAnalysis [3] provides automated conventional psychometric analyses (classical test theory, CTT) alongside the implementation of 1, 2, 3, and 4PL item-response theory (IRT) models and PDF and HTML report options (For review of the alternative 2, 3, and 4PL modeling approaches, see [24]). The development of such open-source cloud-based psychometric software is an emerging interdisciplinary field, defined here as “Shiny Psychometrics”, encompassing (1) psychometrics (e.g., CTT, IRT), (2) data science (i.e., the implementation of algorithms to extract knowledge and insights from structured data), (3) computer science (e.g., cloud-based software development), and (4) learning sciences (e.g., the design of learning innovations for the improvement of instructional methodologies).

The **ShinyItemAnalysis** architecture (https://shiny.cs.cas.cz/ShinyItemAnalysis/) was created from what can be described as an *open-source R Shiny development framework* which enables (a) the immediate integration of efficient cutting-edge statistical and graphical functionality, (b) automatic cloud-based software version updates, and (c) dynamic front-end and comprehensive report rendering capabilities via **rmarkdown**. Considering the general trend and ongoing need in the industry, we chose to develop **autopsych** as a freely available, cloud-based psychometric software.

## 2 Materials and methods

The **autopsych** app point of difference is that it focuses on Rasch modelling, is more accessible to stakeholders, and provides multiple extended functionalities and user-customizations.

### 2.1 Rasch model focus

Like the **ShinyItemAnalysis**, the **autopsych** Shiny app presented here also provides the methodological exposition, analysis, and reproducible reporting of CTT and IRT-based analysis. However, **autopsych** has a particular focus on the PCM Rasch model given its flexibility, practicality to handle different data types, simplicity to provide sufficient item and person statistics (as opposed to 2PL and other IRT models [24]; and the restrictive rating scale model [25]), and broad utility for the measurement of student performance and growth. Masters and Wright [16] provide an eloquent description of the advantages of such models,

The consequence of modelling operating curves to have the same slope is that a unit is defined which enables *all* parameter estimates to be expressed with respect to a common interval scale (which)… supports the quantitative study of growth… (and that) there is no place in these models for schemes which try to assign best weights to items or to response categories [as in the 2PL]. Instead, these models provide coherent and verifiable support for the traditional measurement practice of forming raw scores by counting events. What these models add is control over this traditional (measurement) practice.

[16 p542]

The **autopsych** app adds *ubiquitously accessible practitioner control* over some fundamental aspects of this traditional measurement practice. The app also provides a proof-of-concept for a cloud-based psychometric research platform dedicated to supporting high quality educational assessment and research into the role of individual differences and instructional practice on student learning.

### 2.2 Broad accessibility for developing countries

Even though psychometrics was born more than 300 years ago [26], its modern implementations are not ubiquitous worldwide. There are some countries where the application of psychometric methods is yet to be employed or has only recently been employed. For instance, in 2012, advanced psychometric methods were first applied for the Student Performance Monitoring system for Mathematics [27]. These innovations were made possible with the support of commercially provided psychometric consultancy. However, today there is a shift toward providing open-source software so that individuals and institutions can retain rights to the software and eventually continue to develop the software themselves.

### 2.3 Extended functionality

Specifically, **autopsych** contributes to the growing open-source R Shiny development framework by providing the following extended capabilities:

1. Comprehensive embedded result narration *and* summaries of key outcomes at the start of reports.
2. User-customised item and person flagging systems for identifying anomalous question and person response patterns.
3. Handling and exposition of dichotomous and partial credit data in user-customized Wright maps.
4. User-customized Rasch model constraints and controls.
5. Automated point-biserial orderedness analysis for a detailed examination of utility of polytomous (partial credit) scoring response categories.
6. Single multi-tabbed spreadsheet output providing immediate options for secondary data analysis.
7. Many-facets Rasch analysis for examining differential item functioning (item bias, or ‘invariance’ by groups of interest).
8. Fixed item equating option for mapping students onto different test forms via link items (for developing single ability scales across grades, and for the analysis of growth).
9. A one-way ANOVA tab for examining between-group effects on student ability (e.g., school and gender).
10. Inter-rater reliability analysis option for examining rater consistency in different rater scoring contexts.

The **autopsych** app is built on 31 packages listed in Table A1 in S1 Appendix. In Table A1 in S1 Appendix, for each package, the title, application in autopsych, and the license is also provided. Given that all of the dependent packages have a form of an open-source license, the authors of this paper also decided to ascribe the more recent GNU GPL v3 [28] license to **autopsych**.

### 2.4 Exposition of the autopsych app

In this section, we give an exposition of the **autopsych** UI, customizable, reproducible analysis and reporting options for the uni-dimensional Rasch analysis, many-facets Rasch analysis, Rasch equating, one-way ANOVA, and inter-rater reliability analysis options. To complement this exposition, the **autopsych** app itself can also be accessed here: https://autopsych.shinyapps.io/version_1_0_0/.

### 2.5 Home page and introduction to app

**Fig 1:**
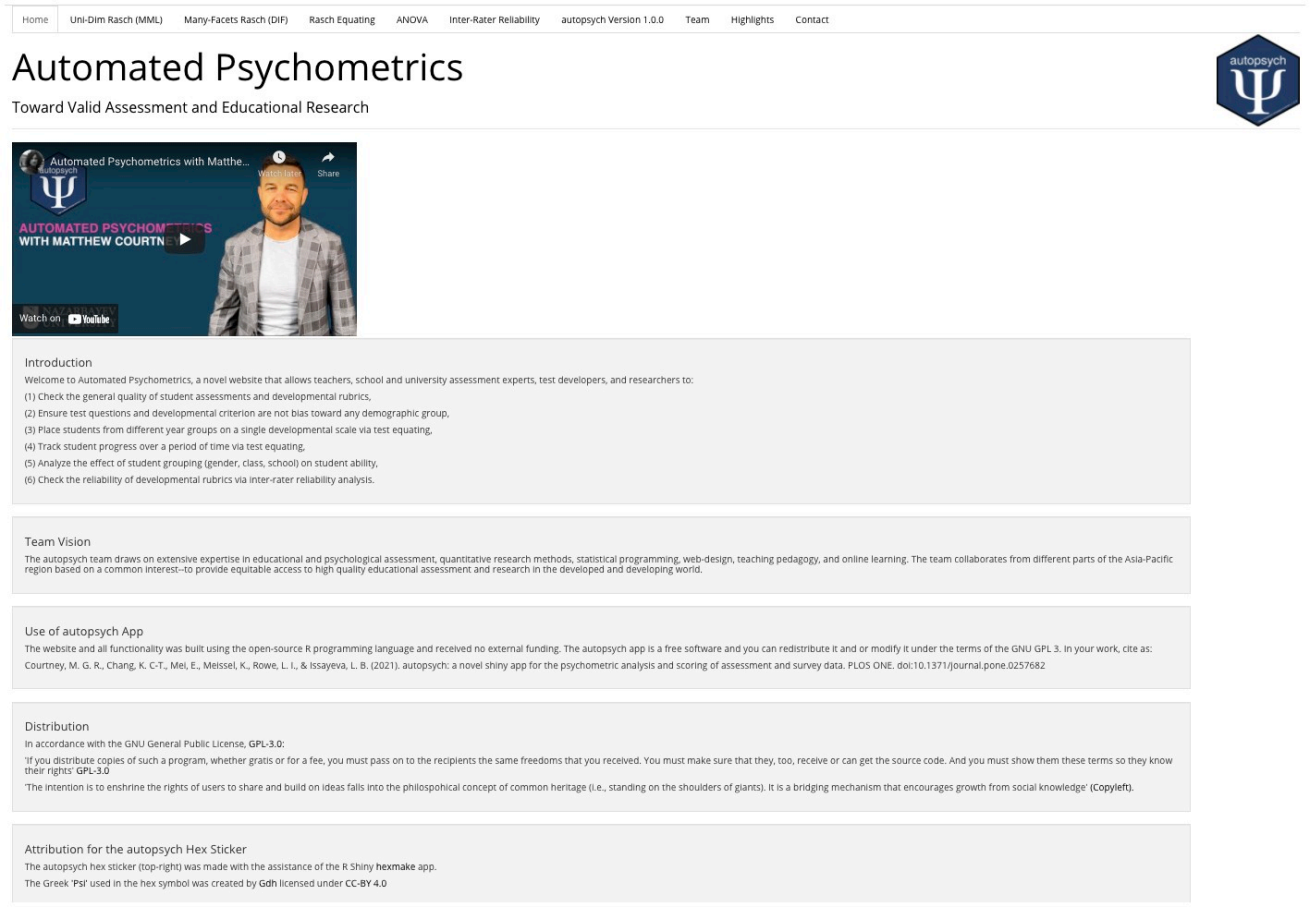
Homepage of autopsych.

The home page of the **autopsych** R Shiny app (Fig 1) introduces six general functions of the app and the co-authors’ vision for making quality assessment and educational research accessible to the developed and developing world.

### 2.6 Uni-dimensional Rasch analysis

**Fig 2:**
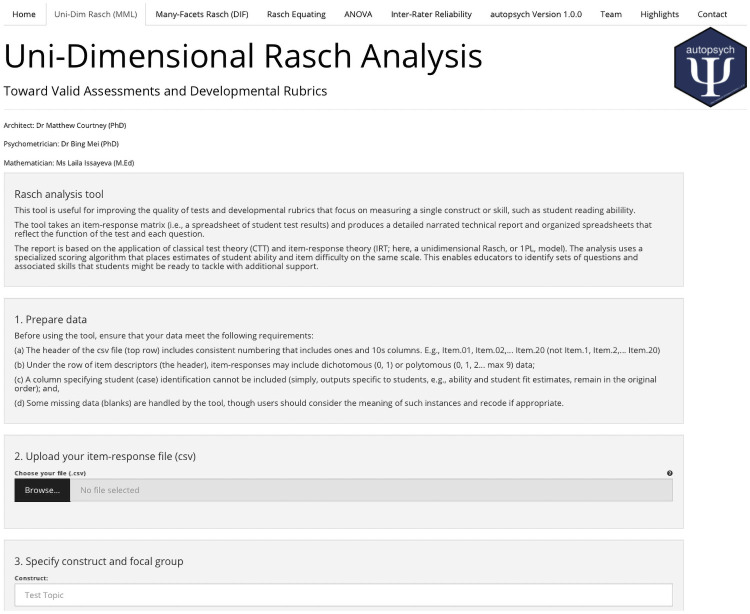
Uni-dimensional Rasch tab.

After a general introduction and instructions on how users should prepare their data, the uni-dimensional Rasch tab (Fig 2) allows users to upload their item-response data. In the current Rasch framework, marginal maximum likelihood estimation (MML) is used. For this analysis, it is assumed that the items constitute a single uni-dimensional construct. For foundational empirical work on various tests of uni-dimensionality in IRT, see Hattie [29]; to implement several modern tests of uni-dimensionality in common statistical programs, see Courtney [30].

After uploading data, users specify the construct (e.g., “Numeracy”) and focal group (e.g., “Auckland students”). Thereafter, users are able to specify the settings for the CTT and Rasch analysis of their data. Users can pre-specify settings for flagging items (panel 4 sliders) and persons (panel 5, “Flag cases…”). Rasch modelling options also include controls over whether or not the model imposes a constraint on cases (persons) or items (questions). Convergence criteria for the model and maximum number of iterations can also be controlled.

Users are also able to specify the graphical settings of the report and make their own notes (perhaps after reviewing the results of an initial report).

**Fig 3:**
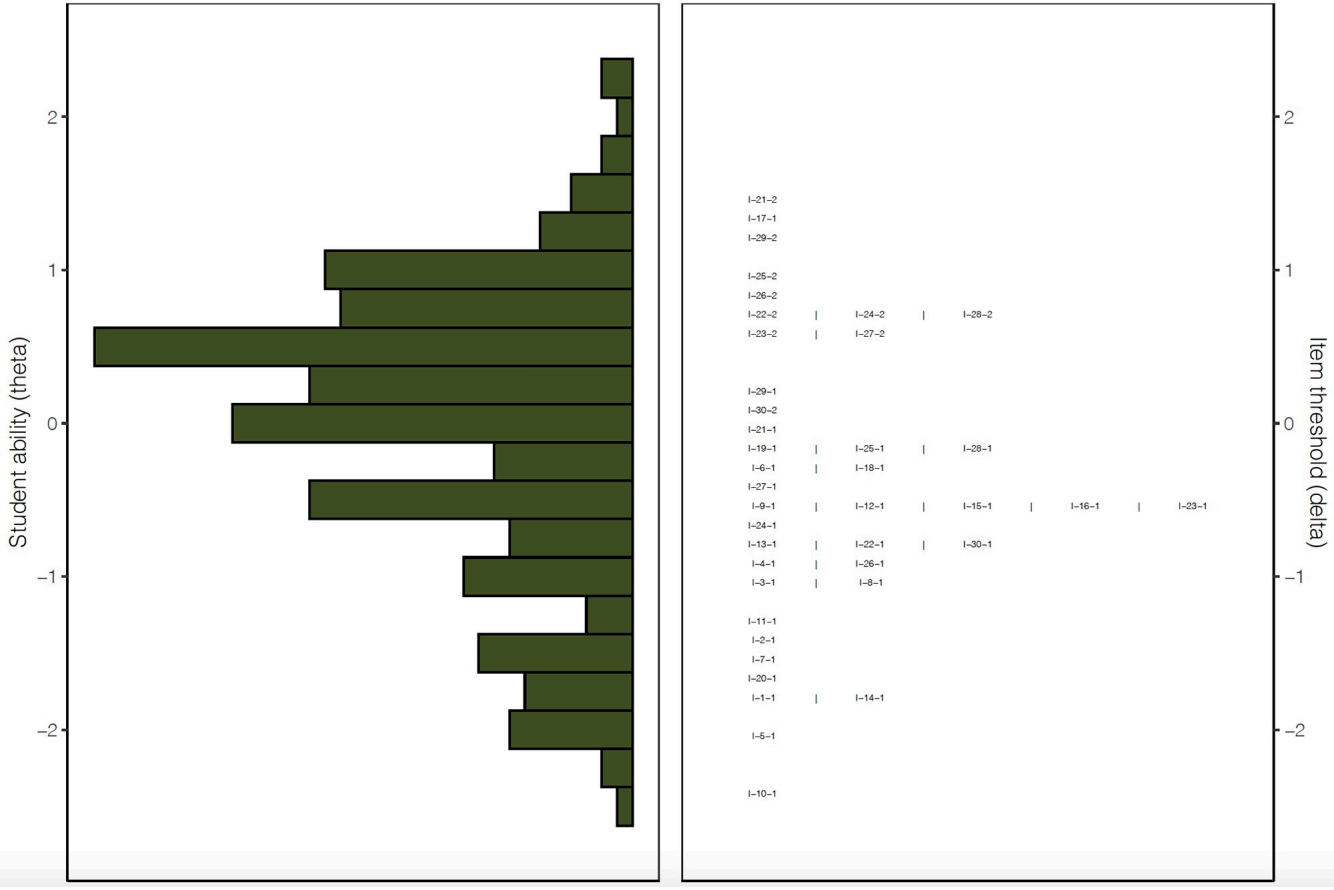
Exposition of dichotomous and partial credit thresholds in Wright map. *Note*. **I-**21-2 represents the item difficulty threshold for the partial credit scores of 0, 1, and 2.

**Fig 4:**
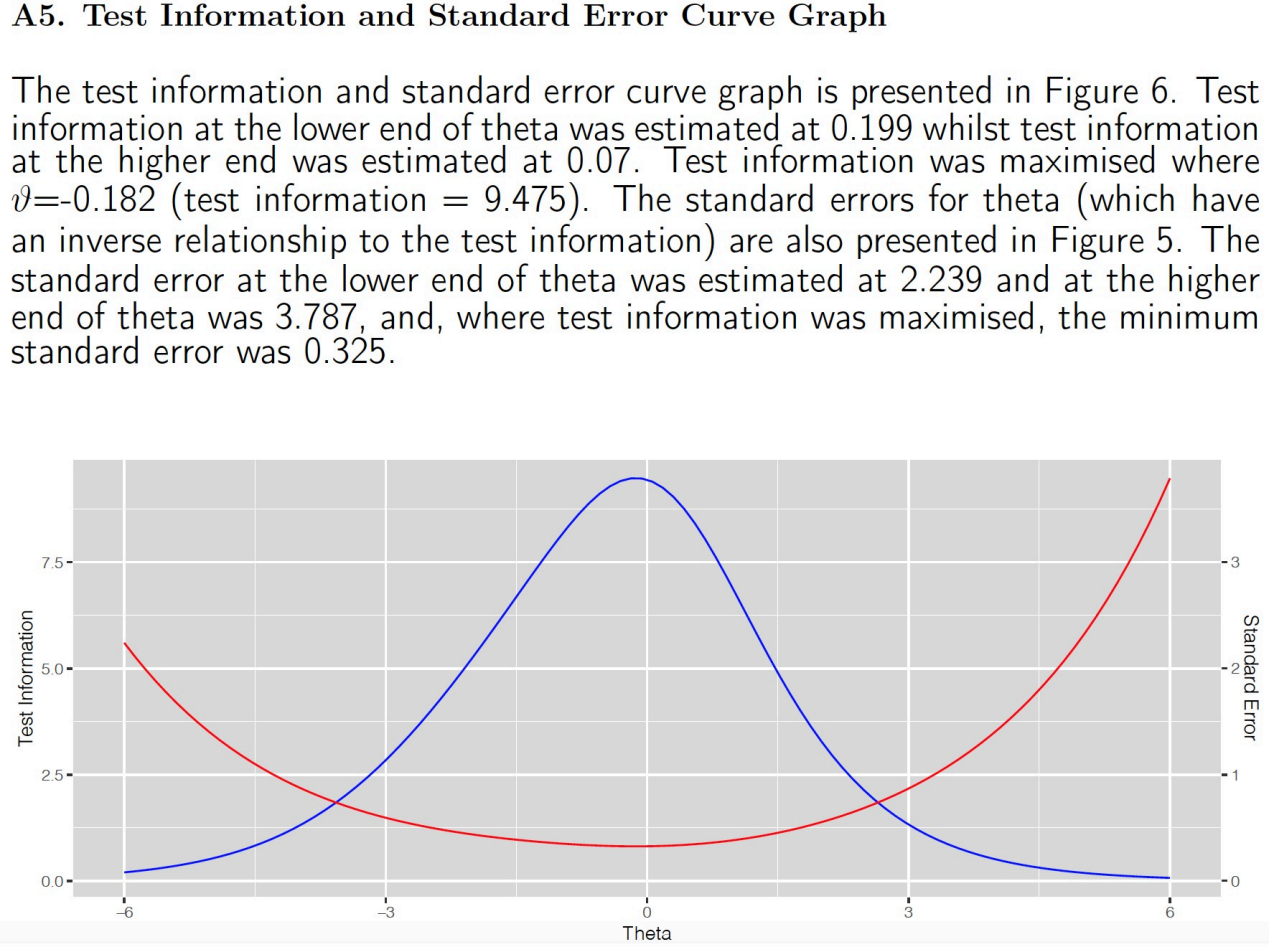
Embedded narration, test information, and standard errors.

The methodology and results in the output technical report (PDF) adapt to account for the user settings. For example, the color and bin width of the Wright map (Fig 3) has been customized in Fig 4. To note, the Wright map provides an exposition of both dichotomous and partial credit scoring thresholds (using the S10 File).

The Wright map suggests that persons and items are matched quite well, though some more difficult item categories could conceivably be created to separate the very top ability students.

**Fig 5:**
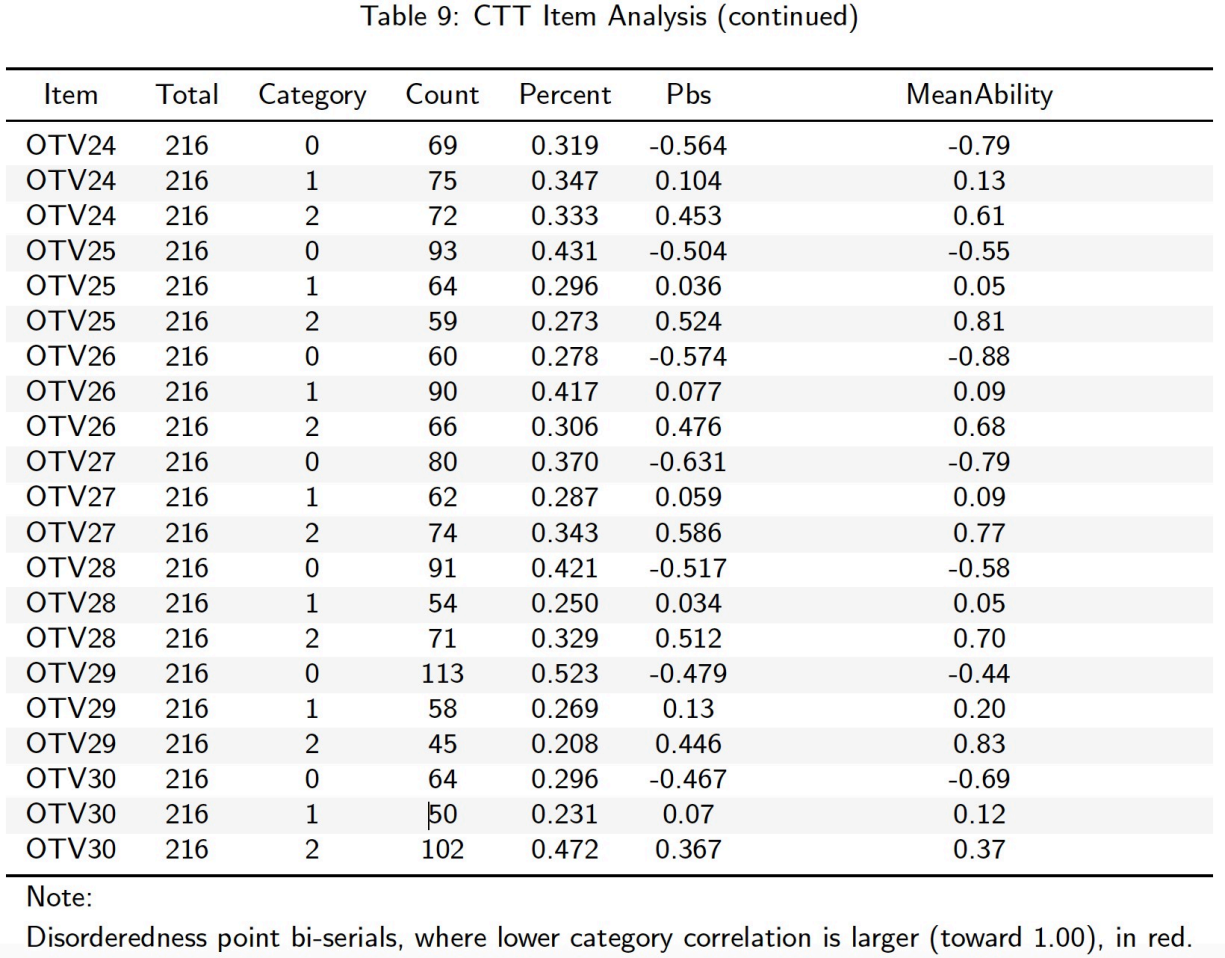
CTT item analysis.

Beyond the illustration of relative student ability and item difficulty via the Wright map, a more formal exposition of test targeting and precision is provided by the test information and standard error (*se*[*θ*_*n*_]) curve graph (Fig 5, Appendix 5 of *output* PDF report). In this case of the dichotomous Rasch model, when *θ*_*n*_ denotes the maximum likelihood estimate,

$$se\left[\theta_{n}\right]={\left[\sum_{i=1}^{I}\text{Pr}\left(X_{ni}=1|\theta_{n},\delta_{i}\right)\left(1-\text{Pr}\left(X_{ni}=1|\theta_{n},\delta_{i}\right)\right)\right]}^{-1}$$ (3)

Conversely, when *δ*_*i*_ denotes the maximum likelihood estimate, *se*[*δ*_*i*_] is given by

$$se\left[\delta_{i}\right]={\left[\sum_{n=1}^{N}\text{Pr}\left(X_{ni}=1|\theta_{n},\delta_{i}\right)\left(1-\text{Pr}\left(X_{ni}=1|\theta_{n},\delta_{i}\right)\right)\right]}^{-1}$$ (4)

With this important information, we can see that the accuracy of student ability estimates may be improved by (a) increasing the number of items in a test, and (b) improving test targeting for all ability levels. Conversely, the precision of item difficulty estimates, and associated resolution into how learning likely unfolds in a particular domain of interest, is improved with a larger and more developmentally-aligned calibrating student sample (see Fig 8, Item Results tab for results of **autopsych** implementation). These simple insights about test information and precision provide continuity between small- and large-scale assessment, and arguably, a more accessible and unifying theory of knowledge and measurement [1 p17].

The slight visual asymmetry in test targeting revealed by the Wright map is supplemented by the test information and standard error curve in Fig 5. The information (provided in Appendix 5 of the *output* PDF report produced for the software user) also includes automated embedded narration vis-à-vis test targeting and accuracy. In this instance, larger standard errors are exhibited at the upper end of the ability spectrum suggesting that the inclusion of slightly more difficult items in subsequent test forms may enable improved precision for higher performing students. For the current test, due to the relatively low number of items (20 dichotomous, 10 polytomous items; S10 File), person standard errors tend to be quite large (min = 0.325 to max 3.787), which may also prompt test designers to include more questions in subsequent tests.

The report also includes the orderedness of item-category point-biserial correlations and mean ability (theta) for each scoring category (e.g., 0, 1, 2). In this instance, the disorderedness in point-biserial correlations is automatically flagged red. However, in this instance, there is no disorderedness, therefore no categories are flagged. As an example, the final three rows of the table give -0.467, 0.07, and 0.367 (Item OTV30 score categories 0, 1, and 2). The correlation between instances of the score 0 (coded “1”) and student theta is, understandably negative (-0.467), while the correlation between instances of the score 2 (coded “1”) is understandably positive (0.367). Consequently, for this item, we would expect the correlation between instances of the middle score category, score 1 (coded “1”), to lie somewhere between -0.467 and 0.367. Therefore, in this case, the item is not flagged. Mean ability for each scoring group is also given in the final column and flagging of disorderedness (reflecting more serious model violations) is also automated.

**Fig 6:**
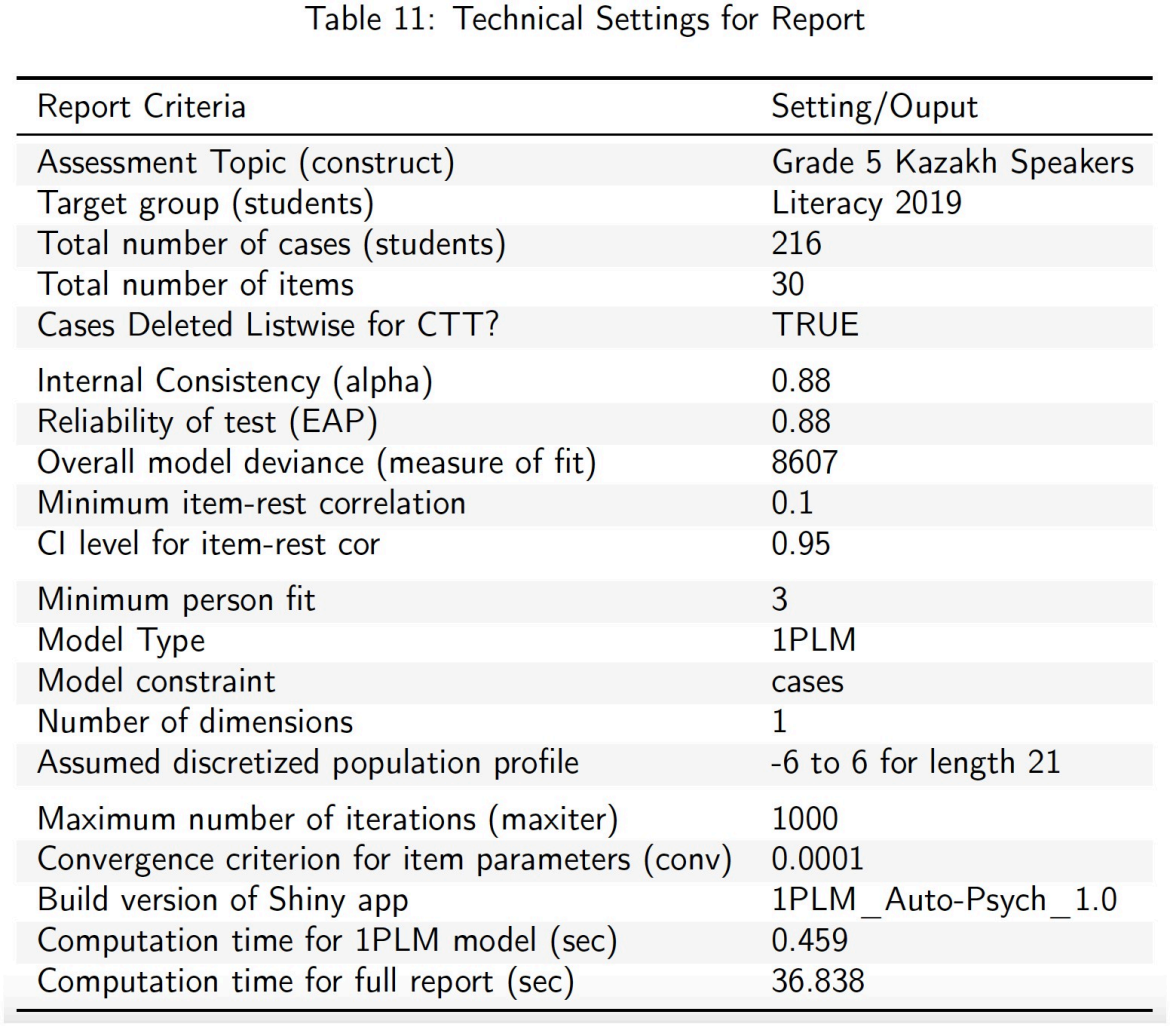
Summary of technical settings and results.

In addition to adaptive methodology and results, a summary of the settings of the report and the general results of the analysis are also provided in an appendix (Fig 6). The output here also includes a measure of overall model deviance with smaller values reflective of improved global model fit [31]. This will be useful when comparing the results of other models in future versions of the app.

**Fig 7:**
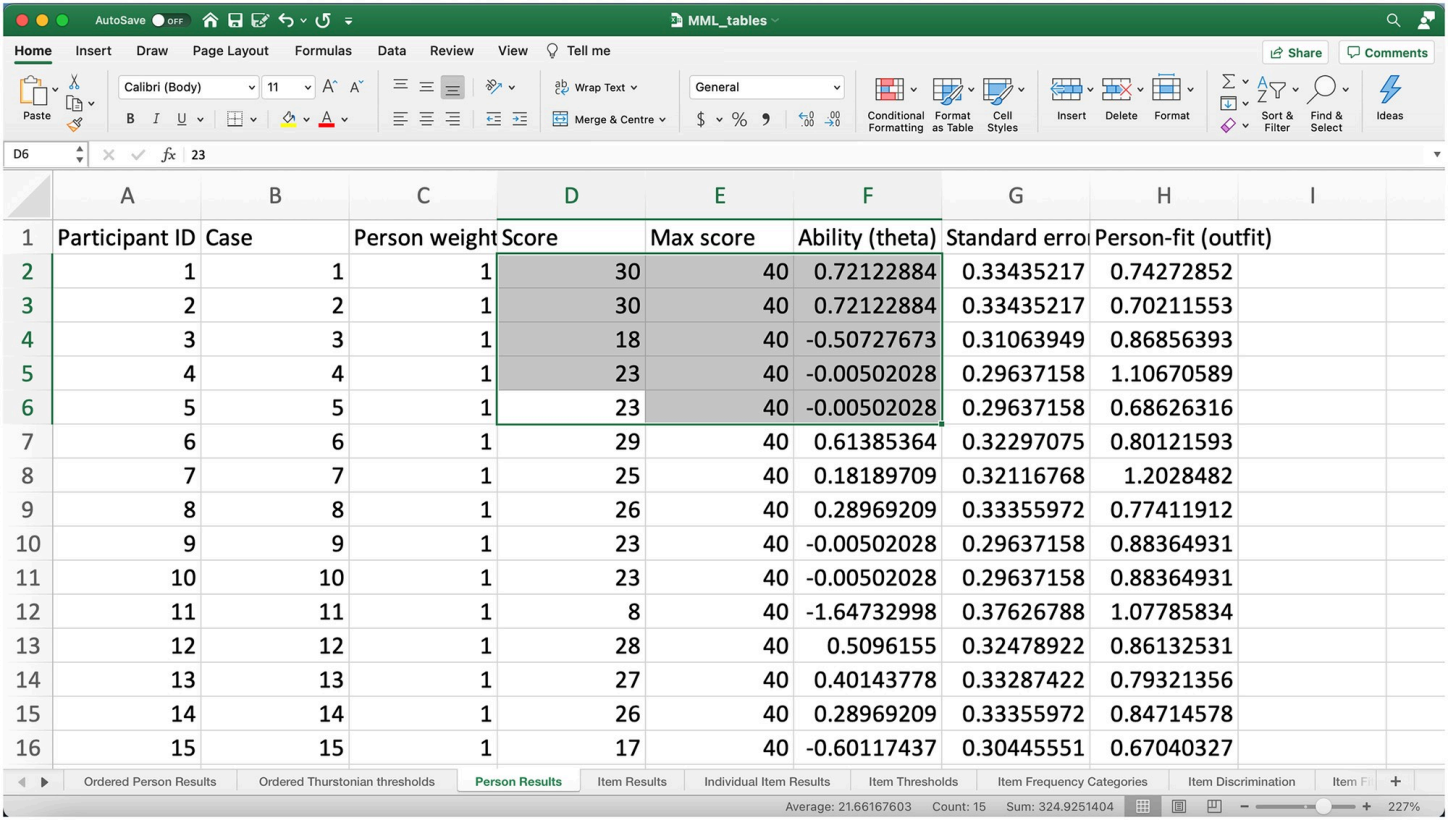
Selection of tabs in output single multi-tabbed spreadsheet. Note. Student Score, Max score, and Ability (theta) is highlighted.

The automatically output.xlsx file includes a collation of all key statistical results from the CTT and Rasch analysis and presents these results via 13 tabs (Fig 7).

Note that total score is a sufficient statistic for theta in the Rasch model (see highlight, Fig 7). Also, the S1 and S2 Files includes tabs for estimates of item difficulty and the full initial item-response matrix. Both of these data become important in the equating procedure illustrated in the Rasch equating section below.

**Fig 8:**
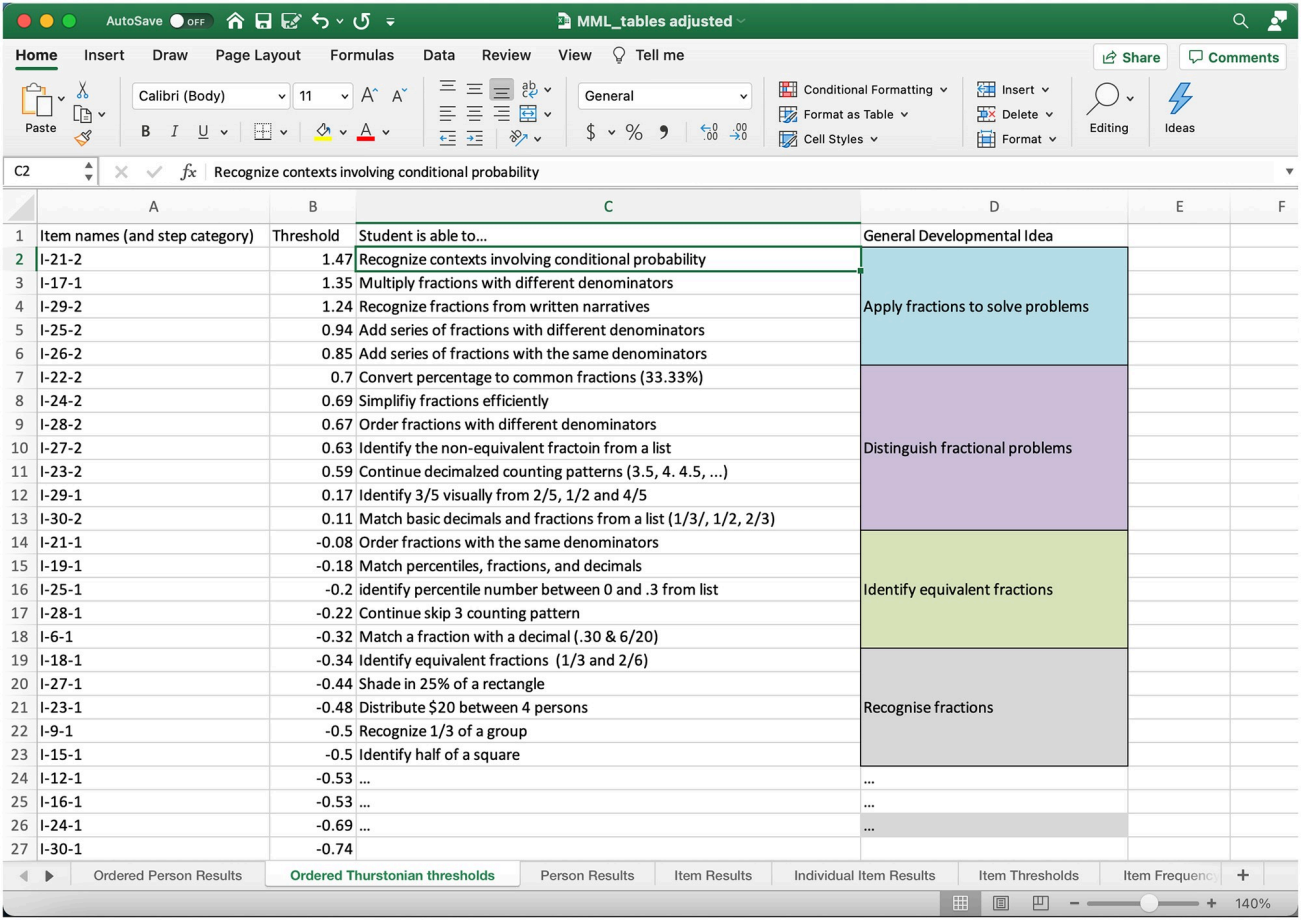
Entering developmental descriptors for item categories. Note. Based upon an analysis of the skills necessary to complete the questions, app users can complete columns C pertaining to the skill associated with each question item of step, and may also like to categorise general developmental ideas, as in column D.

Important to note is that the ability (theta) estimates for each student can then be matched with items or item categories that are comparable. In this instance, students would have a 50:50 odds of completing in the tab to the left, the ordered Thurstonian threshold tab (Fig 8). Essentially, the ordered Thurstonian threshold [32 p170] provides insight into the ordering and reasonable classification of skills in certain domains of learning, and can assist in the development of teaching resources. However, the development of structured teaching resources and curriculum policy becomes more viable when a large number of cases provide more certainty about the actual level of difficulty of skills, and, therefore, how learning likely unfolds in a particular domain (see Wu [33]) on forms of measurement, sampling, and equating errors.

As an example, student 8 who scored 26 out of 30 (theta = 0.29; Fig 8, Participant ID 8), with support from a teacher or peer, may be ready to work toward mastery of continuing decimalized counting patterns (I-23-2, Threshold = 0.59) and, in general, developing in the area of distinguishing fractional problems. Teaching resources and pedagogical approaches for different developmental levels might also be included in another column to the right.

### 2.7 Many-facets Rasch analysis

The many-facets Rasch analysis provides app users with the opportunity to test for item bias against particular student groups (e.g., females), or test for rater effects when the identity of the rater is known. This is done via the addition of an item by group interaction term in the Rasch model. In the case of the dichotomous Rasch model, the facet model applied is given by,

$$P\left(X_{ni}=1|\theta_{n},\delta_{i}\right)=\frac{e^{\left(\theta_{n}-\left(\delta_{i}+G_{g}+D_{gi}\right)\right)}}{1+e^{\left(\theta_{n}-\left(\delta_{i}+G_{g}+D_{gi}\right)\right)}}$$ (5)

where *G*_*g*_ represents the overall group effect on item difficulty, *D*_*gi*_ represents the student group (*g*) by item (*i*) interaction term with Eq 5 basically specifying that the probability of student success on each item depends on an adjustment to the difficulty of the item as a consequence of group membership. For example, if the item favors female students, the *D*_*gi*_ element (representing membership to the female group) will be negative, reflecting the fact that the item is easier for females.

**Fig 9:**
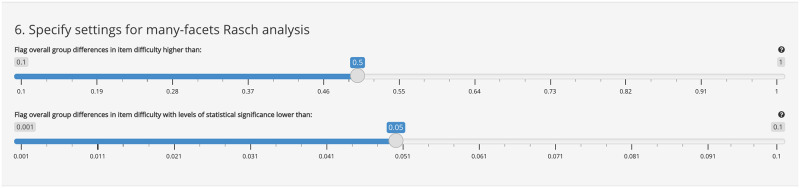
Additional user controls for facets analysis.

Users upload the same type of item-response data to the many-facets Rasch tab though the first column of the csv file should include group membership (e.g., 1 = male, 2 = female). Additional user controls (Fig 9) in the facet tab UI provide options for setting statistical and practical significance of interest for the facets analysis.

**Fig 10:**
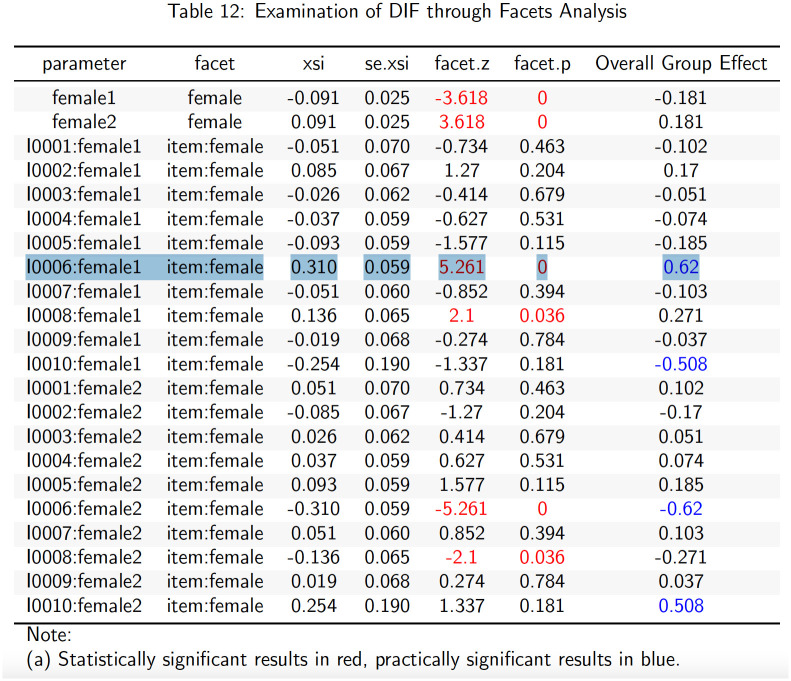
Dynamic tabular results for facet analysis.

**Fig 11:**
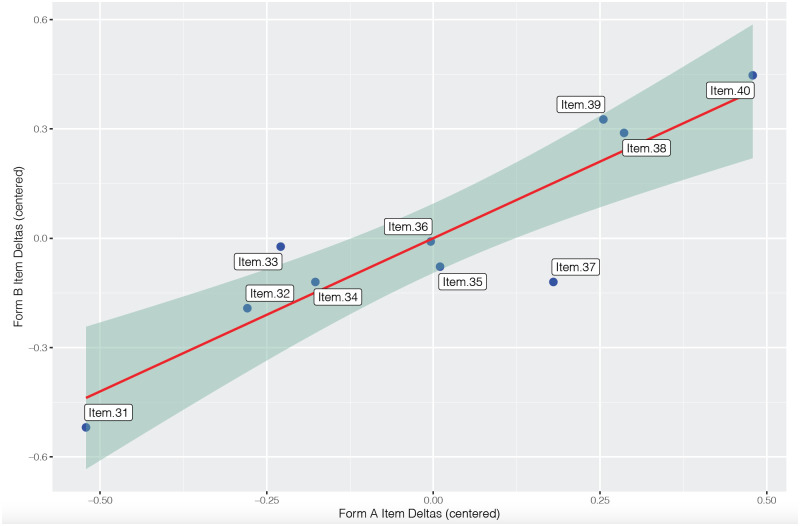
Item difficulty invariance visualization.

In addition to all of the standard PDF and.xlsx outputs provided in the uni-dimensional Rasch MML tab, the Rasch facets tab provides dynamic tabular results (Fig 10) with highlighted elements of interest. In the case of the S5 File data (*N* = 1000), using standard statistical and practical settings, item I0006 is one of the items flagged (blue for practical and red for statistical significance) for being biased against male students (female1 = male, female2 = female; Fig 11). This result may warrant further investigation by the app user. Item I0010 is also flagged for being biased against females (though this effect does not reach statistical significance). A decision on retaining items exhibiting large DIF should be made carefully [32 p217-223].

While the many facets tab helps users identify potentially biased items, the Rasch equating tab enables users to ensure that students who take different tests receive a fair score.

### 2.8 Rasch equating

Test equating is commonly carried out when two (or more) test forms are administered to different groups of students. For example, imagine a 40 item Numeracy test (Form A) is administered to a group of Grade 3 students. At the same time, another 40 item Numeracy test (Form B) is administered to a group of Grade 4 students. In order for both groups of students to receive a fair score on a single scale, the test designers built in some overlap where 10 link items (questions) are delivered in both Test Form A and B assessments (with link items generally a little difficult for Form A students, and easy for Form B students). In order to provide all of the students with a fair score on a single unified scale, one needs to carry out test equating.

Test equating is also carried out when you are tracking student progress across two time periods. Imagine delivering Test Form A at the start of a school year and Test Form B at the conclusion of a school year. Your aim is to provide stakeholders with an understanding of the extent to which each student improved for the given period. To provide students with a fair score for each time period on a unified scale, one needs to carry out test equating.

Here, we make fixed-anchor equating, a common and flexible form of equating, automatically accessible. To illustrate this, we make use of two datasets: S1 and S2 Files. These are simply re-labelled output files from two independent uni-dimensional Rasch analyses. We might imagine that Form A was associated with Grade 3 students while form B was associated with a Grade 4 group (with the link items labelled the same). For this analysis, users simply upload both.xlsx files, complete the settings, then run the analysis. The resultant PDF output produces a visualization to identify item difficulty invariance across test forms (Fig 11).

**Fig 12:**
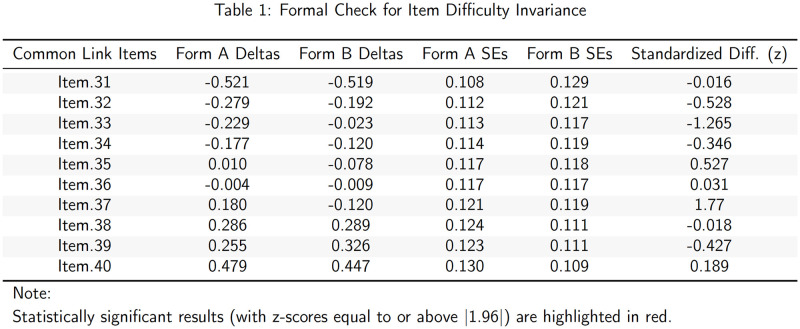
Formal test of link item invariance table.

By inspecting Fig 11, we note that Item 37 may be non-invariant across test forms. For a more formal assessment of invariance, we can assess standardized differences in corresponding items in accordance with,

$$z_{i}=\frac{\delta_{i}-\overset{´}{\delta_{i}}}{\sqrt{se[\delta_{i}{]}^{2}+se[\overset{´}{\delta_{i}}{]}^{2}}}$$ (6)

where, *z*_*i*_ is the standardised delta difference for each corresponding item, *δ*_*i*_ is the item parameter for item *i* in Test Form A, and ${\overset{´}{\delta }}_{i}$ is the corresponding item parameter for item *i* in Test Form B. Note that $\sum_{i=1}^{L}\left(\delta_{i}-{\overset{´}{\delta }}_{i}\right)=0$, where *L* is the total number of link items. Fig 12 illustrates the output associated with the formal check.

Further, the standardized error of equating can be calculated in accordance with,

$$\varepsilon =\frac{\sqrt{\frac{\sum_{i=1}^{L}{\left(\delta_{i}-{\overset{´}{\delta }}_{i}\right)}^{2}}{L-1}}}{\sqrt{L}}$$ (7)

where *ε* is the equating error, *L* is the number of link items, *δ*_*i*_ is the item parameter for item *i* in Test Form A, and ${\overset{´}{\delta }}_{i}$ is the corresponding item parameter for item *i* in Test Form B. Using the provided, the PDF report states that the standard error of equating is 0.042 logits.

**Fig 13:**
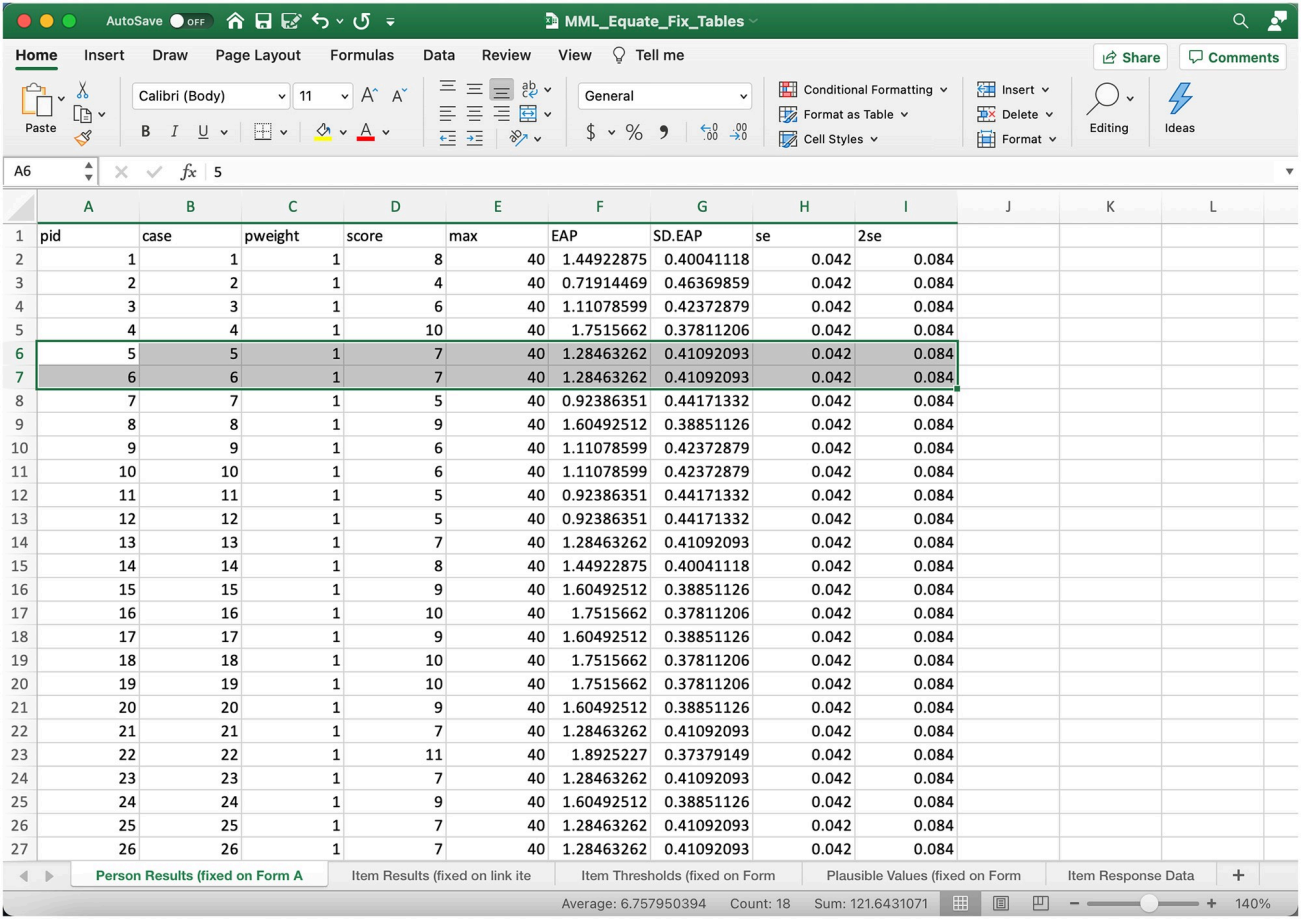
Output.xlsx file from fixed equating procedure.

**Fig 14:**
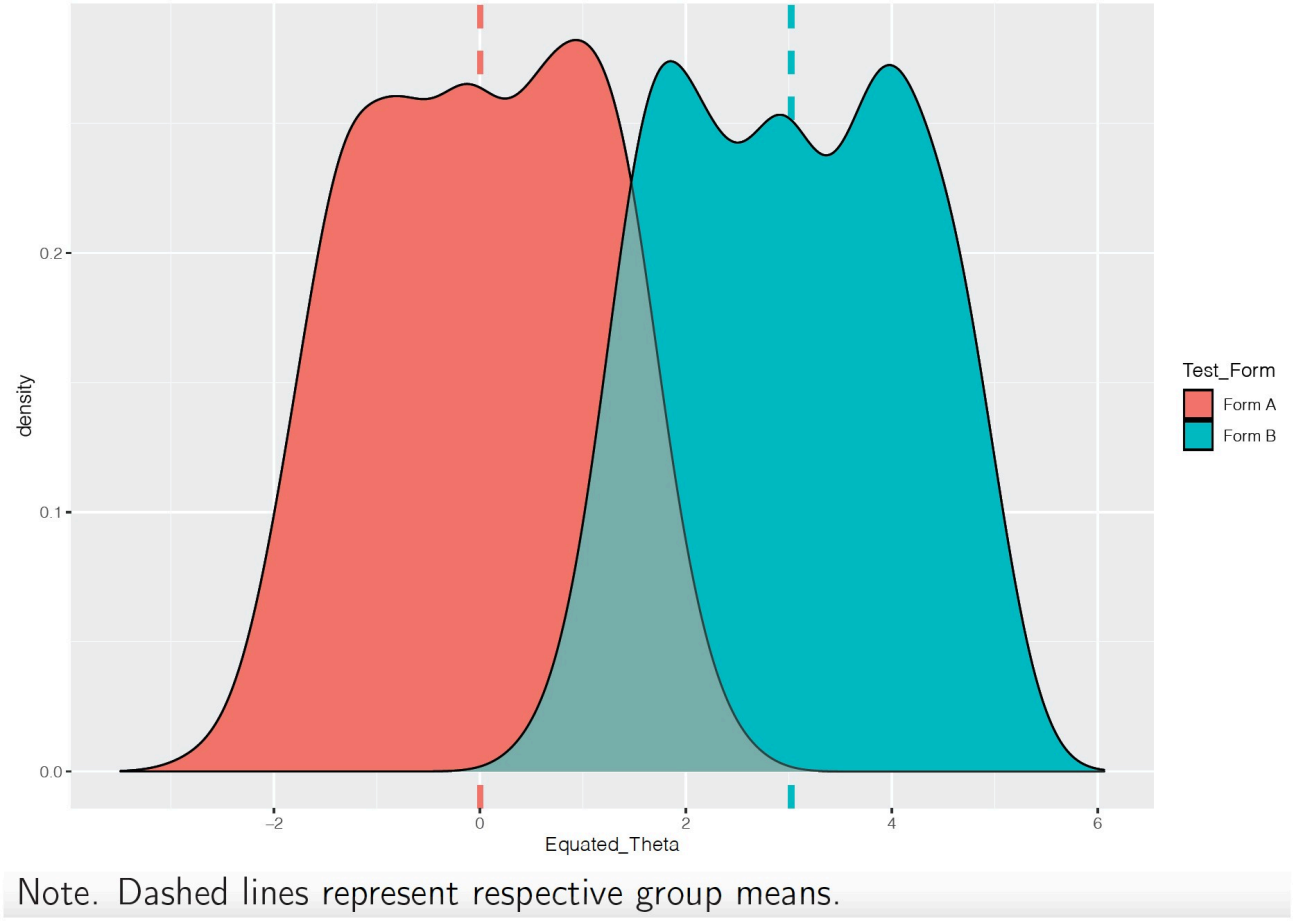
Density plot for equated student ability (theta).

The output.xlsx file (Fig 13) includes the person ability and item difficulty estimates for Test Form B students (fixed onto test Form A). As a consequence of test equating, the student ability estimates from both test forms can now be compared across test forms. Fig 14 provides a density plot for the Form B student ability estimates (aqua) equated with the ability estimates from Test Form A (red). This gives insight into the distributional properties of student ability form both test forms, and the breadth of student ability measured by both test forms.

### 2.9 One-way ANOVA

**Fig 15:**
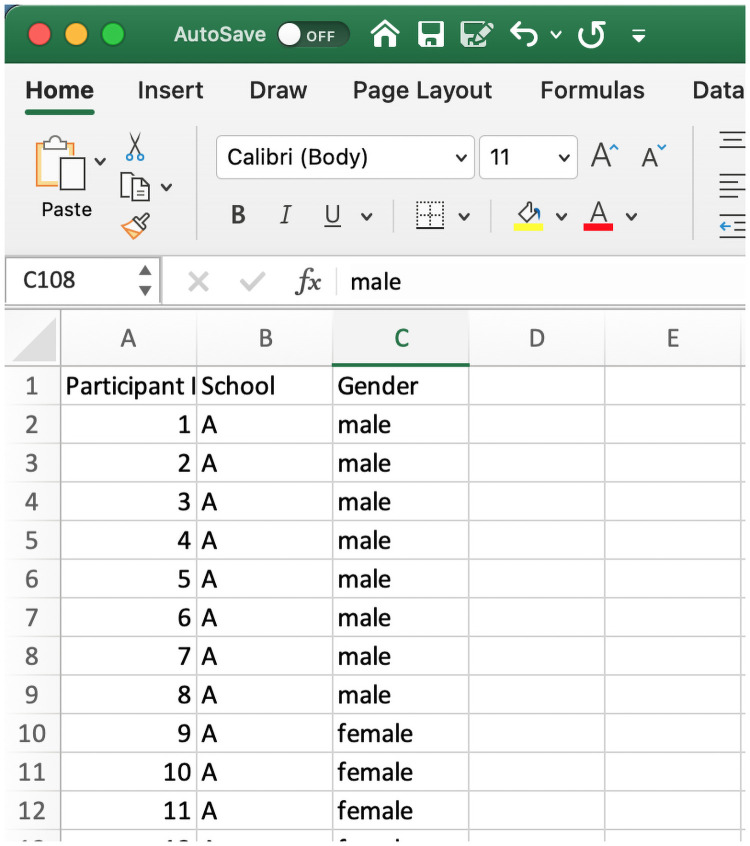
The Test_ANOVA_Group.xlsx file.

The app also provides an automated one-way ANOVA to examine the effect of student grouping on the student ability outcome of interest. The tab takes two data inputs: the first is the.xlsx output from the uni-dimensional Rasch tab; the second contains an.xlsx file containing the grouping variable(s) of interest. The one-way ANOVA function takes the ability theta variable from the person tab (example data: S4 File) and another file containing the grouping variables (S3 File). The S3 File (Fig 15) contains any number of grouping variables with each element corresponding to the same participant in the S4 File dataset.

**Fig 16:**
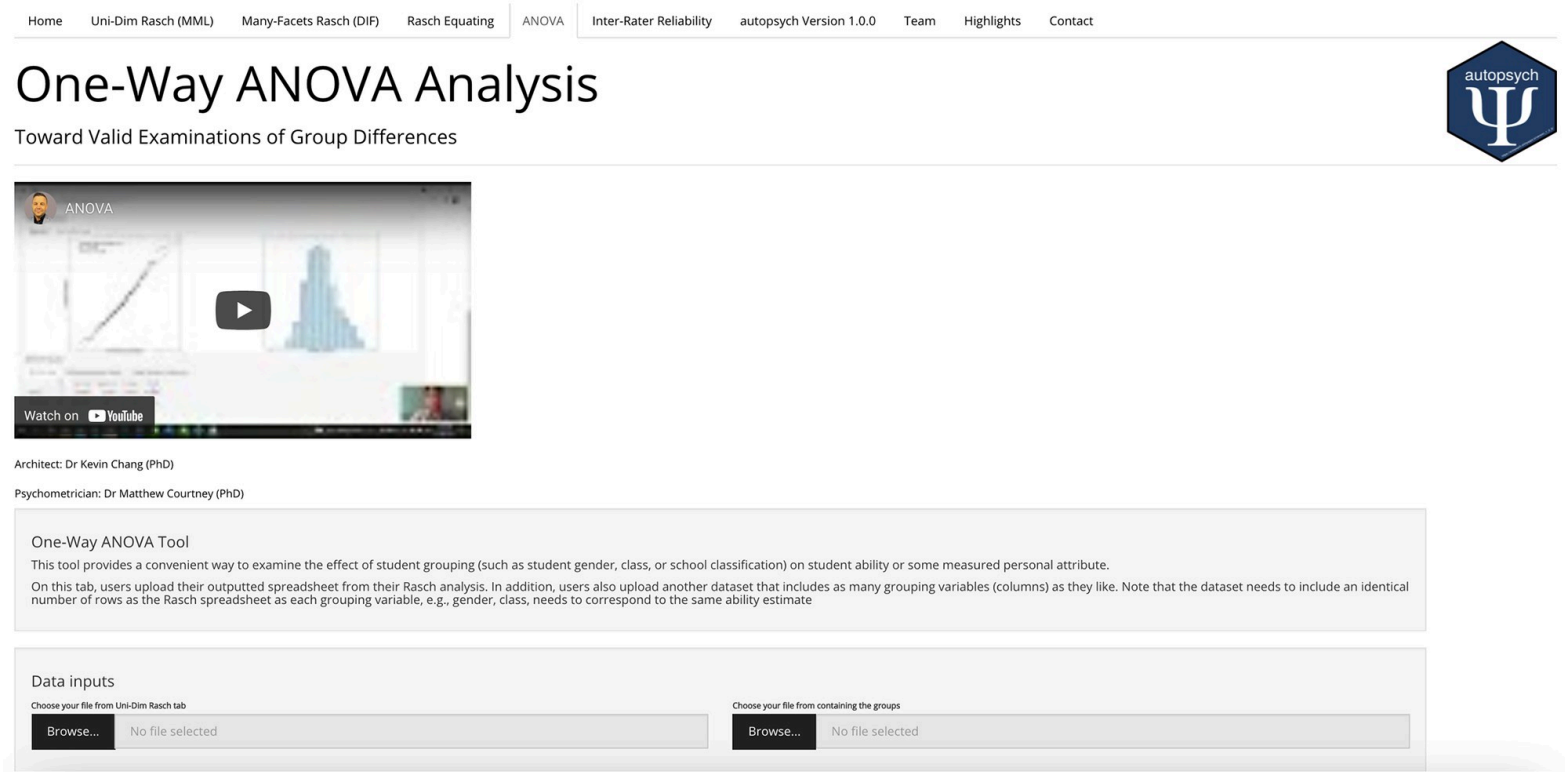
One-way ANOVA function.

The ANOVA function provides users with immediate results and options to examine the effects of the student grouping variable of interest on the student ability (theta). Fig 16 provides an example of the output boxplots in addition to normality checks, ANOVA tables, Estimated marginal means, and Tukey pairwise comparisons.

### 2.10 Inter-reliability analysis

**Fig 17:**
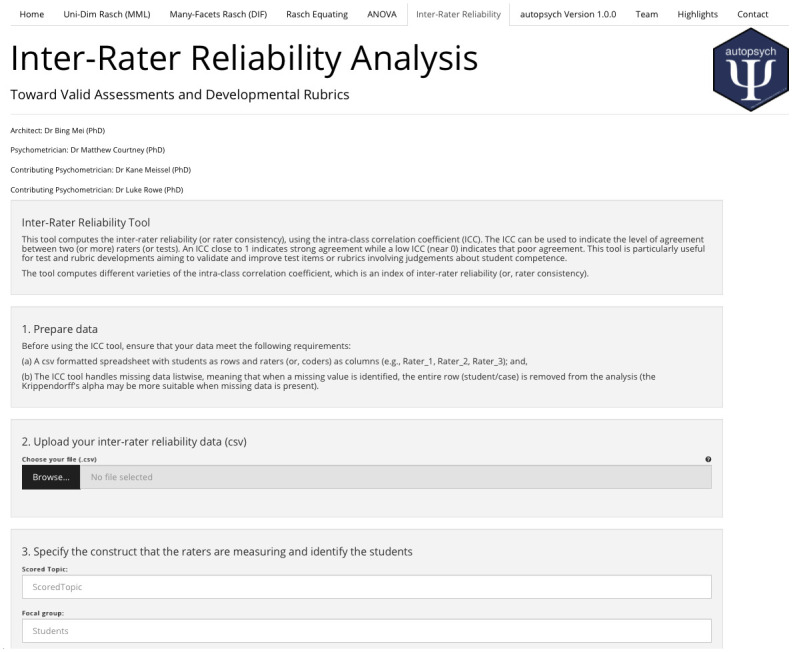
Inter-rater reliability function.

Student products, such as essays and other written or oral performances, are often judged by multiple raters. One way to determine the consistency of marking (and, perhaps the utility of the instrument) is to estimate the level of inter-rater reliability from a set of data. One versatile way to determine rater consistency is to use the intra-class correlation coefficient. The **autopsych** app also provides this option (Fig 17).

**Fig 18:**
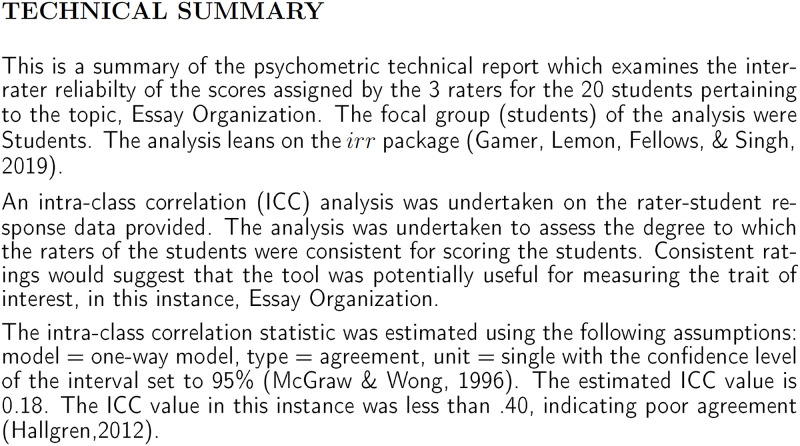
Inter-rater technical summary example as provided by autopsych.

Depending on the data conditions and focus of the analysis, users may specify the model, type, unit of analysis, and confidence interval for the intra-class correlation (ICC) statistic. Using the provided S8 File, and standard settings, a rendered report is produced. The report provides a technical summary (Fig 18) with all results in the first section while a more detailed description of the methodology adopted by the user is given in the latter sections.

### 3. App performance testing

App testing involved the use of simulated item-response data with different conditions using an *I* by *N* design (with *I* = items and *N* = student sample size). Four total item number conditions included 20, 40, 60, and 80 dichotomous items reflective of quite short school-based assessments to more prolonged external assessments. Sample sizes included 50 (an approximate minimum for exploratory work), 500, 10,000, 50,000, 100,000, to 500,000 (similar to PISA). This meant that a total of 24 conditions were tested: 4(item) by 6(person) conditions (see S2 Appendix of this document for code used to simulate these data). Performance testing was not carried out for the many-facets and equating tab as these involved minor procedural extensions to the uni-dimensional tab and likely similar. In addition, the ANOVA and IRR functionality were not tested as these procedures are far less computationally intensive.

The first tests were run locally on a 2.2 GHz Quad-Core Intel Core i7 processor with 16GB 1600 MHz DDR3 Ram using an Intel Iris pro 1536 MB graphics card. The second tests (Table 1, in brackets) were run on the online version: https://autopsych.shinyapps.io/version_1_0_0/ (with 8GB instance size, datasets with *I* = 60 *N* = 50,000 ran successfully, while larger datasets could only be processed locally with 16GB ram). All performance tests were run with standard settings. Results in minutes are presented in Table 1. Results suggest that the online version when *N* was less than 10,000, reports take less than two minutes to produce, though computation time balloons to over an hour and a half with 80 items and 500,000 students.

**Table 1:** Uni-dimensional Rasch report computational time (in minutes).

Item/N Conditions	*N* = 50	*N* = 500	*N* = 10,000	*N* = 50,000	*N* = 100,000	*N* = 500,000
*I* = 20	0.36 (0.25)	0.39 (0.21)	0.62(0.52)	2.20(1.62)	4.18	47.24
*I* = 40	0.63 (0.39)	0.67 (0.37)	1.16(.84)	3.48(2.65)	7.23	61.23
*I* = 60	0.50 (0.48)	0.56 (0.52)	1.64(1.23)	5.35(3.85)	11.97	86.54
*I* = 80	1.17 (0.63)	1.23 (0.68)	1.97(1.52)	6.77	13.47	94.80

*Note*. Test times not in brackets pertain to locally run shiny app; test times in brackets pertain to those done online with the R Shiny app; all tests completed successfully; see Appendix B in S2 Appendix to replicate item-response data.

## 4. Conclusions

The **autopsych** app promotes and makes accessible high quality educational assessment and related research into student learning. The platform makes use of CTT and Rasch-based modelling to (a) provide continuity between classroom-based and large-scale assessments, (b) make information about test quality immediately accessible, (c) provide teachers and learners with immediate feedback about what might be useful to teach/learn next, (d) support the establishment of unified test forms, and (e) enable the examination of the effects of student grouping on student ability. Though the app is certainly not comprehensive—point-and-click software will always be a step ahead due to significant licensing costs that help support development but also reduce access. However, the app provides a proof of concept for a more sophisticated and intelligent assessment system that places learners and the teachers as “the primary consumers and benefactors of the information derived from assessment” [34 p17].

### 4.1 Future directions

The app has the potential to evolve in a number of directions. The app’s utility lies in its capacity to make powerful psychometric procedures, those typically only available proprietarily, ubiquitously accessible. Therefore, it is envisaged that future developments and derivative works will focus on expanding the app’s Rasch-based and related functionality to account for nested data conditions common to educational research.

A fundamental rule in technology says that whatever *can* be done *will be* done.

[35 p46]

The convergence between the disciplines of psychometrics, data science, computer science, and the learning sciences is inevitable. However, these improved efficiencies also carry inherent risk. Potential blind user reliance on outputs and strict adherence to rules-of-thumb need to be countered providing users with not only source code, but also clear methodological expositions and referenced reading and learning material. The app **autopsych** app makes an attempt to do this.

### 4.2 Final thoughts

It should be noted that quality assessment starts at the early design stage with a foundational understanding of the various forms of validity, and the construction of items and developmental criteria that adequately, and representatively, sample the content area to be measured [2]. Part 1 of this important book [2] presents key concepts associated with validation, precision and errors of measurement, and fairness in testing—insights about tests worth making universally accessible. To this end, an *open-source R Shiny development framework* provides a state-of-the-art ecosystem for the on-going co-creation of a suite of user-friendly tools that contribute positively toward an expanded psychometric tool-box. Finally, it is the authors’ view that the more ubiquitous application of improved measurement practice—beyond the fields of educational and health assessment [36] to the psychological sciences—may offer an important way out of the replicability crisis [37]. Early examples provide a useful food for thought [38].

## Supporting information

S1 Appendix (DOCX)

S2 Appendix (DOCX)

S1 File (XLSX)

S2 File (XLSX)

S3 File (XLSX)

S4 File (XLSX)

S5 File (CSV)

S6 File (CSV)

S7 File (CSV)

S8 File (CSV)

S9 File (CSV)

S10 File (CSV)
