# Supplementary material for: autopsych: An R Shiny tool for the reproducible Rasch analysis, differential item functioning, equating, and examination of group effects
Source: PLoS One. 2021 Oct 11;16(10):e0257682. doi: 10.1371/journal.pone.0257682 (PMC8505029; doi:10.1371/journal.pone.0257682)
Supplement: S1 Appendix — (DOCX) [file pone.0257682.s001.docx]

**S1 Appendix**

| **Table A1. Dependent packages for autopsych.** | | | |
| --- | --- | --- | --- |
| **R package** | **Title** | Application in **autopsych** | **License** |
| **Data manipulation** | | | |
| **dplyr** [1] | A Grammar of Data Manipulation | Data preparation for conditional tables | GPL v3 |
| **plyr** [2] | The Split-Apply-Combine Strategy for Data Analysis | Group means for fixed equating | MIT |
| **openxlsx** [3] | Read, Write and Edit xlsx Files | Collation and Manipulation of Excel files and tabs | MIT |
| **reshape2** [4] | Reshaping Data with the reshape Package | Data manipulation of heatmap matrix | MIT |
| **janitor** [5] | Simple tools for examining and cleaning dirty data | Data management in the ANOVA tab | MIT |
| **magrittr** [6] | A forward-pipe operator for R. | Enables piping in ANOVA user interface (UI) module | MIT |
| **Psychometrics** | | | |
| **CTT** [7] | Classical Test Theory Functions | Various CTT applications | GPL v2 |
| **Hmisc** [8] | Harrell Miscellaneous | Item correlation matrices | GPL v2 |
| **NCmisc** [9] | Miscellaneous Functions for Creating Adaptive Functions and Scripts | z-to-p functions for facets analysis | GPL v2 |
| **psychometric** [10] | Applied Psychometric Theory | Correlation confidence intervals | GPL v2 |
| **TAM** [11] | Test Analysis Modules | Rasch analyses | GPL v2 |
| **irr** [12] | Various coefficients of interrater reliability and agreement | Inter-rater reliability analysis | GPL v2 |
| **lmerTest** [13] | Tests in linear mixed effects models | ANOVA test tab | GPL v2 |
| **emmeans** [14] | Estimated Marginal Means, aka Least-Squares Means | ANOVA test tab | GPL v3 |
| **s20x** [15] | s20x: Functions for University of Auckland Course STATS 201/208 Data Analysis | ANOVA test tab | GPL v |

| **Table A1. Dependent Packages for autopsych. (*Continued...*)** | | | |
| --- | --- | --- | --- |
| **R package** | **Title** | Application in **autopsych** | **License** |
| **Graphics** | | | |
| **scales** [16] | Scale functions for visualization | Tailored scales for graphs | MIT |
| **ggplot2** [17] | Elegant graphics for data analysis | Base functions for all graphs | GPL v3 |
| **ShinyItemAnalysis** [18] | … for Teaching Psychometrics and to Enforce Routine Analysis of Educational Tests | Base functions for dichotomous/polytomous Wright maps | GPL v3 |
| **cowplot** [19] | Streamlined Plot Theme and Plot Annotations for 'ggplot2' | Enables grids in Wright maps | GPL v2 |
| **ggrepel** [20] | Automatically Position Non-Overlapping Text Labels with 'ggplot2' | Enables non-overlap of item difficulty labels when checking for invariance | GPL v3 |
| **Tables, embedded narration, and rendering** | | | |
| **knitr** [21] | A General-Purpose Package for Dynamic Report Generation in R | Dynamic tabular reporting | GPL v1 |
| **kableExtra** [22] | Construct Complex Table with 'kable' and Pipe Syntax | Pre-specification of cell format in tables | MIT |
| **english** [23] | Translate integers into English | Assists embedded narration and APA formatting of numbers | GPL v2 |
| **xtable** [24] | Export Tables to LaTeX or HTML | Conversion of R object correlation matrix to xtable object to be printed as latex | GPL v2 |
| **Shiny app and rendering** | | | |
| **rmarkdown** [25] | Dynamic Documents for R | Rendering of tailored PDF documents | GPL v3 |
| **shiny** [26] | Web Application Framework for R | UI, server, development for **autopsych** | GPL v3 |
| **shinyjs** [27] | Easily Improve the User Experience of Your Shiny Apps in Seconds | Enables implementation of java script in UI | MIT |
| **shinythemes** [28] | Themes for Shiny | Enables pre-specified themes for UI; “cosmo” used herein | GPL v3 |
| **shinyBS** [29] | Twitter Bootstrap Components for Shiny | Adds “tool tip” to shiny inputs in UI | GPL v3 |
| **bsplus** [30] | Adds Functionality to the R Markdown + Shiny Bootstrap Framework | Enables notes to become readable as users hover over question marks | MIT |
| **shinyWidgets** [31] | Custom Inputs Widgets for Shiny | Enables enhanced functionality, i.e., setting background image in UI | GPL v3 |

**References (S1 Appendix)**

1. Wickham H, François R, Henry L, Müller K. dplyr: A grammar of data manipulation. R package version 1.0.2. 2020. Available from: https://CRAN.R-project.org/package=dplyr

2. Wickham H. The split-apply-combine strategy for data analysis. J Stat Softw. 2011; 40(1): 1-29.

3. Schauberger P, Walker A. openxlsx: Read, write and edit xlsx Files. R package version 4.2.3. 2020. Available from: [https://CRAN.R-project.org/package=openxlsx](https://cran.r-project.org/package=openxlsx)

4. Wickham H. Reshaping data with the reshape package. J Stat Softw. 2007; 21(12): 1-20.

5. Firke S. janitor: Simple tools for examining and cleaning dirty data. R package version 2.0.1. 2020. Available from: [https://CRAN.R-project.org/package=janitor](https://cran.r-project.org/package=janitor)

6. Milton S, Wickham H, Henry L. magrittr: A forward-pipe operator for R. R package version 2.0.1. 2020. Available from: [https://CRAN.R-project.org/package=magrittr](https://cran.r-project.org/package=magrittr)

7. Willse JT. CTT: Classical test theory functions. R package version 2.3.3. 2018. Available from: [https://CRAN.R-project.org/package=CTT](https://cran.r-project.org/package=CTT)

8. Harrell FE Jr, Dupont C. Hmisc: Harrell Miscellaneous. R package version 4.4-2. 2020. Available from: [https://CRAN.R-project.org/package=Hmisc](https://cran.r-project.org/package=Hmisc)

9. Cooper N. NCmisc: Miscellaneous functions for creating adaptive functions and scripts. R package version 1.1.6. 2018. Available from: [https://CRAN.R-project.org/package=NCmisc](https://cran.r-project.org/package=NCmisc)

10. Fletcher TD. psychometric: Applied Psychometric Theory. R package version 2.2. 2010. Available from: [https://CRAN.R-project.org/package=psychometric](https://cran.r-project.org/package=psychometric)

11. Robitzsch A, Kiefer T, Wu M. TAM: Test analysis modules. R package version 3.5-19. 2020. Available from: [https://CRAN.R-project.org/package=TAM](https://cran.r-project.org/package=TAM)

12. Gamer M, Lemon J, Fellows I, Singh P. irr: Various coefficients of interrater. R package version 0.84.1. 2019. Available from: [https://CRAN.R-project.org/package=irr](https://cran.r-project.org/package=irr)

13. Kuznetsova A, Brockhoff PB, Christensen RHB. lmerTest package: Tests in linear mixed effects models. Journal of Statistical Software. 2017; 82(13): 1-26.

14. Lenth R. emmeans: Estimated marginal means, aka least-squares means. R package version 1.5.2-1. 2020. Available from: [https://CRAN.R-project.org/package=emmeans](https://cran.r-project.org/package=emmeans)

15. Balemi A, Chandra D, Curran J, Deppa B, Forster M, McArdle B, et al. s20x: Functions for University of Auckland Course STATS 201/208 Data Analysis. R package version 3.1-30. 2021. Available from: [https://CRAN.R-project.org/package=s20x](https://cran.r-project.org/package=s20x)

16. Wickham H, Seidel D. scales: Scale functions for visualization. R package version 1.1.1. 2020. Available from: https://CRAN.R-project.org/package=scales

17. Wickham H. ggplot2: Elegant Graphics for Data Analysis. Springer-Verlag: New York; 2016.

18. Martinková P, Drabinová A. ShinyItemAnalysis for teaching psychometrics and to enforce routine analysis of educational tests. R J. 2018 Dec; 10(2): 503-515.

19. Wilke CO. cowplot: Streamlined plot theme and plot annotations for 'ggplot2'. R package version 1.1.0. 2020. Available from: https://CRAN.R-project.org/package=cowplot

20. Slowikowski K. ggrepel: Automatically position non-overlapping text labels with 'ggplot2'. R package version 0.9.0. 2020. Available from: [https://CRAN.R-project.org/package=ggrepel](https://cran.r-project.org/package=ggrepel)

21. Xie Y, Vogt A, Andrew A, Zvoleff A, Simon A, Atkins A, et al. knitr: A general-purpose package for dynamic report generation in R. R package version 1.30. 2020. Available from: <https://cran.r-project.org/web/packages/knitr/index.html>

22. Zhu H. kableExtra: Construct complex table with 'kable' and pipe syntax. R package version 1.3.1. 2020. Available from: [https://CRAN.R-project.org/package=kableExtra](https://cran.r-project.org/package=kableExtra)

23. Fox J, Venables B, Damico A, Salverda AP. english: Translate Integers into English. R package version 1.2-5. 2020. Available from: [https://CRAN.R-project.org/package=english](https://cran.r-project.org/package=english)

24. Dahl DB, Scott D, Roosen C, Magnusson A, Swinton J. xtable: Export Tables to LaTeX or HTML. R package version 1.8-4. 2019. Available from: [https://CRAN.R-project.org/package=xtable](https://cran.r-project.org/package=xtable)

25. Allaire JJ, Xie Y, McPherson J, Luraschi J, Ushey K, Atkins A, et al. rmarkdown: Dynamic documents for R. R package version 2.5. 2020. Available from: <https://rmarkdown.rstudio.com>

26. Chang W, Cheng J, Allaire JJ, Xie Y, McPherson J. shiny: Web Application Framework for R. R package version 1.5.0. 2020. Available from: [https://CRAN.R-project.org/package=shiny](https://cran.r-project.org/package=shiny)

27. Attali D. shinyjs: Easily improve the user experience of your Shiny apps in seconds. R package version 2.0.0. 2020. Available from: [https://CRAN.R-project.org/package=shinyjs](https://cran.r-project.org/package=shinyjs)

28. Chang W. shinythemes: Themes for Shiny. R package version 1.1.2. 2018. Available from: [https://CRAN.R-project.org/package=shinythemes](https://cran.r-project.org/package=shinythemes)

29. Bailey E. shinyBS: Twitter bootstrap components for Shiny. R package version 0.61. 2015. Available from: [https://CRAN.R-project.org/package=shinyBS](https://cran.r-project.org/package=shinyBS)

30. Lyttle I. bsplus: Adds Functionality to the R Markdown + Shiny Bootstrap Framework. R package version 0.1.2. 2020. Available from: [https://CRAN.R-project.org/package=bsplus](https://cran.r-project.org/package=bsplus)

31. Perrier V, Meyer F, Granjon D. shinyWidgets: Custom inputs widgets for shiny. R package version 0.5.4. 2020. Available from: [https://CRAN.R-project.org/package=shinyWidgets](https://cran.r-project.org/package=shinyWidgets)
