# Supplementary material for: autopsych: An R Shiny tool for the reproducible Rasch analysis, differential item functioning, equating, and examination of group effects
Source: PLoS One. 2021 Oct 11;16(10):e0257682. doi: 10.1371/journal.pone.0257682 (PMC8505029; doi:10.1371/journal.pone.0257682)
Supplement: S2 Appendix — (DOCX) [file pone.0257682.s002.docx]

**S2 Appendix**

#### Simulating data for app performance testing in R ####

rm(list=ls())

# 20 conditions: # I = 20, 40, 60 80; # N = 50, 500, 10000, 50000, 100000, 500000

# E.g., Condition 1: I=20, N=50

I <- 20

N <- 50

ability <- seq(-2, 2,length=N)

difficulty <- seq(-2, 2, length=I)

expected.perf <- plogis( outer( ability , difficulty , "-" ) )

set.seed(0123)

resp1 <- 1 * ( expected.perf > matrix( runif( N*I ) , nrow=N , ncol=I ) )

colnames(resp1) <- paste("I" , sprintf("%02d", 1:I), sep="")

write.csv(resp1, "Sim.I20.N50.csv", row.names = F)
